# The Effect of Sacubitril/Valsartan on Supraventricular and Ventricular Arrhythmias in Patients With Heart Failure

**DOI:** 10.1111/anec.70081

**Published:** 2025-04-15

**Authors:** Alireza Arzhangzadeh, Mohammad Hossein Nikoo, Majid Haghjoo, Fatemeh Rasekh, Shayan Shojaei, Asma Mousavi, Salma Nozhat, Roozbeh Narimani‐Javid, Helia Bazroodi, Sana Neisi, Mitra Mojibpour, Mohammad Abedini, Saghi Eslamzadeh, Hamed Bazrafshan Drissi, Sasan Shafiei

**Affiliations:** ^1^ Department of Cardiology Shiraz University of Medical Sciences Shiraz Iran; ^2^ Cardiovascular Research Center, Department of Cardiovascular Medicine Shiraz University of Medical Sciences Shiraz Iran; ^3^ Rajaie Cardiovascular Medical and Research Center Iran University of Medical Sciences Tehran Iran; ^4^ Student Research Committee, School of Medicine Shiraz University of Medical Science Shiraz Iran; ^5^ Cardiac Primary Prevention Research Center, Cardiovascular Diseases Research Institute Tehran University of Medical Sciences Tehran Iran; ^6^ Tehran Heart Center, Cardiovascular Diseases Research Institute Tehran University of Medical Sciences Tehran Iran; ^7^ Research Center for Advanced Technologies in Cardiovascular Medicine, Cardiovascular Diseases Research Institute Tehran University of Medical Sciences Tehran Iran

**Keywords:** arrhythmias, CRT‐D, heart failure, ICD, Sacubitril/Valsartan

## Abstract

**Background:**

Patients with heart failure with reduced ejection fraction (HFrEF) frequently experience electrical disturbances, such as ventricular or atrial fibrillation (AF). Sacubitril/Valsartan (SV) therapy has been linked to lower rates of mortality, ventricular tachycardia (VT), and ventricular fibrillation (VF), with decreased reliance on implantable cardioverter‐defibrillator (ICD) therapy. However, studies on the antiarrhythmic effects of SV in patients with ICD or cardiac resynchronization therapy defibrillator (CRT‐D) devices are limited. This study aimed to evaluate the impact of SV therapy on antiarrhythmic pacing, defibrillation shock occurrences, and the burden of ventricular arrhythmias in patients with HFrEF who have ICD or CRT‐D devices.

**Method:**

This study was conducted at a HF outpatient clinic involving patients with HFrEF treated with SV. Primary outcomes included the incidence of VT, VF, non‐sustained VT (NsVT), supraventricular tachycardia (SVT), and related interventions such as antiarrhythmic pacing (ATP) and defibrillation shocks.

**Result:**

A total of 181 HFrEF patients completed at least 12 months of follow‐up, with a mean age of 63.39 ± 12 years; 36.5% were male, and 60.8% had an ICD. Device interrogation revealed a significant reduction in VF incidents (7 vs. 15, *p* = 0.025) and a decrease in the combined outcome of VT and VF (17 vs. 24, *p* = 0.047). The need for ICD interventions such as ATP and shocks also significantly decreased following the initiation of SV therapy (10 vs. 24, *p* = 0.012).

**Conclusion:**

SV therapy significantly reduces the incidence of cardiac arrhythmias, particularly VT and VF, while decreasing the need for clinical interventions related to implanted devices.

## Graphical abstract

Patients undergoing SV therapy had significantly less VTVF events and associated clinical interventions. This effect could lead to less possibility of lethal arrhythmia and sudden cardiac death.

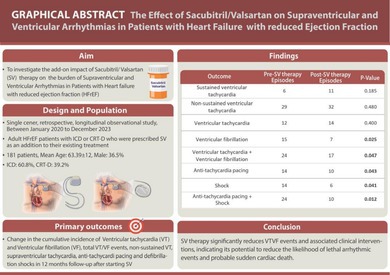

**Abbreviations**
ACEIs: angiotensin‐converting enzyme inhibitors
AF: atrial fibrillation
ARBs: angiotensin receptor blockers
ARNI: angiotensin receptor‐neprilysin inhibitor
ATP: antiarrhythmic pacing
CABG: coronary artery bypass graft
CKD: chronic kidney disease
CRT‐Ds: cardiac resynchronization therapy‐defibrillators
DM: diabetes mellitus
ESC: European Society of Cardiology
GLS: global longitudinal strain
HF: Heart failure
HFrEF: heart failure and reduced ejection fraction
HR: heart rate
HTN: hypertension
ICDs: implantable cardioverter‐defibrillators
LAD: left atrial diameter
LAVI: left atrial volume index
LVEDD: left ventricular end‐diastolic diameter
LVEDVI: Left ventricular end diastolic volume indices
LVEF: left ventricular ejection fraction
LVESD: left ventricular end‐systolic diameter
LVESVI: left ventricular end systolic volume indices
MI: myocardial infarction
NSVT: non‐sustained ventricular tachycardia
NYHA: New York Heart Association
NYHA: New York Heart Association
pro‐BNP: pro‐B‐type natriuretic peptide
PVC: premature ventricular contractions
RA: right atrial
RAAS: renin–angiotensin–aldosterone system
RCTs: randomized control trials
RVOT: right ventricular outflow tract
SCD: sudden cardiac death
SD: standard deviation
SV: Sacubitril/Valsartan
SVT: Supraventricular tachycardia
TAPSE: Tricuspid annular plane systolic excursion
VAs: ventricular arrhythmias
VF: ventricular fibrillation
VT: ventricular tachycardia

## 1 Introduction

Heart failure (HF) is a complex clinical condition characterized by the heart's inability to effectively pump blood to satisfy the body's metabolic demands (Doenst et al. 2013). Along with typical signs and symptoms, including fluid accumulation, shortness of breath, and persistent fatigue, patients with HF frequently encounter electrical disturbances (Schwinger 2021). It is estimated that around one third are present with ventricular conduction blockages, while approximately one third to 50% have atrial fibrillation (AF). Moreover, nearly half of these patients may experience premature ventricular contractions (PVCs) (Prinzen et al. 2022). Both AF and ventricular arrhythmias can lead to serious complications, including thromboembolic events linked to AF and sudden cardiac death (SCD) resulting from sustained ventricular tachycardia (VT) or ventricular fibrillation (VF) (Huizar et al. 2019). To prevent SCD associated with arrhythmias, therapeutic interventions such as implantable cardioverter‐defibrillators (ICDs) and cardiac resynchronization therapy‐defibrillators (CRT‐Ds) are recommended (Varga et al. 2024). These interventions have been shown to effectively reduce both short‐term and long‐term mortality rates in patients with HF and reduced ejection fraction (HFrEF) (Schrage et al. 2019). Nonetheless, pharmacological treatment remains a fundamental strategy for the prevention and management of arrhythmias, primarily due to economic factors and the broader applicability of these established treatment modalities (Wang et al. 2022).

Studies investigating Sacubitril/Valsartan (SV) therapy have shown significant benefits for patients with HF, particularly those classified within NYHA classes II–IV (Jaffuel et al. 2021). The findings suggested that SV therapy, irrespective of the left ventricular ejection fraction (LVEF), significantly reduced mortality rates in HF patients (McMurray et al. 2014). SV therapy has also been shown to decrease the incidence of VT and VF, resulting in lower usage of ICD therapy (Rohde et al. 2020). Furthermore, it has been demonstrated that SV add‐on therapy in CRT‐D patients enhanced therapeutic responses, alleviated symptoms, and diminished hospital readmissions related to HF, AF, and ventricular arrhythmias (Russo et al. 2022). Positive outcomes have also been observed in ICD patients receiving SV therapy, with a notable reduction in the occurrence of VT and atrial arrhythmias (Russo et al. 2020). Despite these promising findings, the underlying mechanisms by which SV may confer antiarrhythmic benefits remain incompletely understood. Some studies suggest that the modulation of neurohormonal activity and sympathetic tone may contribute to this effect (Carnagarin et al. 2018). However, these outcomes may also result from the ability of SV to enhance ventricular remodeling while simultaneously lowering the risk of ventricular arrhythmias compared to traditional angiotensin‐converting enzyme inhibitors (ACEIs) and angiotensin receptor blockers (ARBs) (Russo et al. 2020; Curtain et al. 2022; Tsai et al. 2021). Additionally, SV therapy appears to promote improvements in atrial remodeling and reduce the incidence of atrial arrhythmias (Li et al. 2022; Suo et al. 2019).

Nevertheless, there are few studies regarding the antiarrhythmic effects of SV in patients receiving ICD therapy or CRT‐D as secondary prevention. In this study, we aimed to investigate the add‐on impact of SV therapy on the occurrence of antiarrhythmic pacing (ATP), defibrillation shock, and the burden of ventricular arrhythmias in HF patients with ICD and CRT‐D devices.

## 2 Materials and Methods

### 2.1 Study Design and Population

This study was a monocentric, retrospective, longitudinal observational study conducted at a HF outpatient clinic in Shiraz, Iran. The study adhered to the principles outlined in the Declaration of Helsinki concerning research involving human subjects. Patients diagnosed with HFrEF who were treated with SV between January 2020 to December 2023 were screened for inclusion. The inclusion criteria for this analysis were as follows: (A) Patients aged 18 years or older with HFrEF defined as an ejection fraction of 40% or less; (B) the presence of an ICD or CRT‐D and interrogation assessment of the device every 3 months; (C) prior treatment with an individually optimized dose of either ARBs or ACEIs since HF diagnosis; and (D) prior treatment with threepillars (maximum tolerated dosages of approved beta‐blocker and mineralocorticoids such as spironolactone and eplerenone, and fixed dosage of sodium‐glucose cotransporter 2 such as empagliflozin and dapagliflozin) which completed with SV as the final pillar of fantastic four (Docherty et al. 2022). The indications for ICD and CRT‐D implantation adhered to the guidelines set forth by the European Society of Cardiology (ESC) (Brignole et al. 2013). Moreover, we included ICD patients with > 99% ventricular sensing and CRT‐D patients with > 95% biventricular pacing in order to reduce the confounding factor of diminished LVEF. Patients were excluded from this study if they met any of the following criteria: (1) simultaneous device implantation and initiation of SV therapy; (2) new device implantation or modification during SV therapy; (3) discontinuation of SV therapy due to adverse effects such as hypotension, depressed kidney function, allergic reaction, or hyperkalemia; (4) occurrence of acute cardiac decompensation; (5) classification as NYHA class IV HF with unstable clinical conditions and refractory symptoms; (6) prior treatment with other antiarrhythmic drugs except digoxin; or (7) occurrence of refractory ventricular tachyarrhythmia, refractory arrhythmia, and recurrent ICD shock which lead to VT ablation.

### 2.2 Collection of Baseline Characteristics

Demographic data were collected retrospectively by reviewing patient medical records. Information regarding past medical history, including hypertension (HTN), diabetes mellitus (DM), dyslipidemia, chronic kidney disease (CKD), prior myocardial infarction (MI), AF, and previous coronary artery bypass graft (CABG), and physical examinations, including heart rate (HR) and blood pressure records, were obtained. The New York Heart Association (NYHA) classification was used to assess functional capacity. The etiologies of HF were classified as ischemic or nonischemic. This study was observational and retrospective; thus, all evaluations were conducted as part of standard clinical care rather than for research purposes. Device recordings were obtained from each patient for a total of 24 months, encompassing 12 months before and 12 months after the initiation of SV treatment. This design ensures that the follow‐up period is equal for both pre‐ and posttreatment evaluations, allowing for a direct comparison of event occurrences. Additionally, it is worth noting that up‐titration of SV was performed every 6 weeks to reach the maximum tolerated dose. Relevant data from objective evaluations of cardiac devices and echocardiograms were collected during routine follow‐up visits both prior to and following SV treatment. Furthermore, we evaluated the duration of SV therapy, any adverse events leading to treatment discontinuation, potential changes in antiarrhythmic medications, and occurrences of therapies that could influence arrhythmia risk based on the last follow‐up visit. All participants maintained SV treatment for a minimum of 12 months, which enabled us to compare data collected before SV therapy with outcomes observed 12 months after treatment initiation. Based on the last follow‐up visit, the duration of SV treatment, any adverse events leading to discontinuation, potential changes in antiarrhythmic medications, and occurrences of therapies that could alter arrhythmia risk were evaluated.

### 2.3 Primary and Secondary Outcomes

The primary outcome of this study was to evaluate the cumulative incidence of VT, VF, total VTVF events, non‐sustained ventricular tachycardia (NsVT, defined as ≥ 4 beats < 30 s), supraventricular tachycardia (SVT), encompassing atrial fibrillation and atrial high‐rate episodes, and the administration of appropriate therapies such as ATP and defibrillation shocks through follow‐ups of 3 months during 12 months. Patients also underwent echocardiography at both the initiation of SV therapy and during follow‐up assessments. For secondary outcomes, changes in left ventricular end‐diastolic diameter (LVEDD) and LVEF were calculated.

### 2.4 Echocardiographic Measures

We conducted a standard transthoracic echocardiography to collect information on the LV structure and function, following the guidelines of the American Society of Echocardiography (Mitchell et al. 2019). Digital echocardiographic data are stored locally on a hard disk. Echocardiographic measurements were investigated by five independent observers (AA, MHN, MH, SN, and HBD). LVEDD was measured in two‐dimensional targeted M‐mode echocardiographic tracings in the parasternal long‐axis view. LVEF was measured using a modified Simpson's biplane method. An experienced cardiologist, blinded to the study, reviewed all measurements.

### 2.5 Device Interrogating

Cardiac device interrogation and event analysis were performed by an electrophysiologist blinded to our study.

### 2.6 Device Programming Protocol

Standardized device programming was done in all patients at our high‐volume university electrophysiology lab. ICD and CRT device programming was tailored on an individual basis based on predefined heart rate zones that are described below:

- VF Zone (≥ 200 bpm): ATP during charging followed by high‐energy shocks; detection interval is 30–40 to avoid inappropriate therapy.
- Fast VT Zone (170–199 bpm): Prolonged detection (e.g., 60 s); ATP as initial treatment with repeated sequences.
- VT Zone (< 170 bpm): Monitored primarily; ATP utilized if therapy was indicated.

SVT discrimination algorithms were enabled in all devices. The first shock energy was set at maximum output, and lead integrity monitoring was turned on. This protocol was intended to maximize arrhythmia termination and minimize unnecessary intervention.

### 2.7 Statistical Analysis

For quantitative variables and qualitative variables, we reported means ± standard deviation (SD) and percentages or frequencies, respectively. The Wilcoxon signed‐rank test was employed to assess the difference in quantitative variables before and after treatment with SV. To evaluate the relationships between all variables and changes in quantitative measures, we utilized univariable and multivariable linear regression analyses. Statistical analyses were conducted using SPSS version 22 software, with a significance level set at 5%.

## 3 Results

### 3.1 Study Population

**FIGURE 1:**
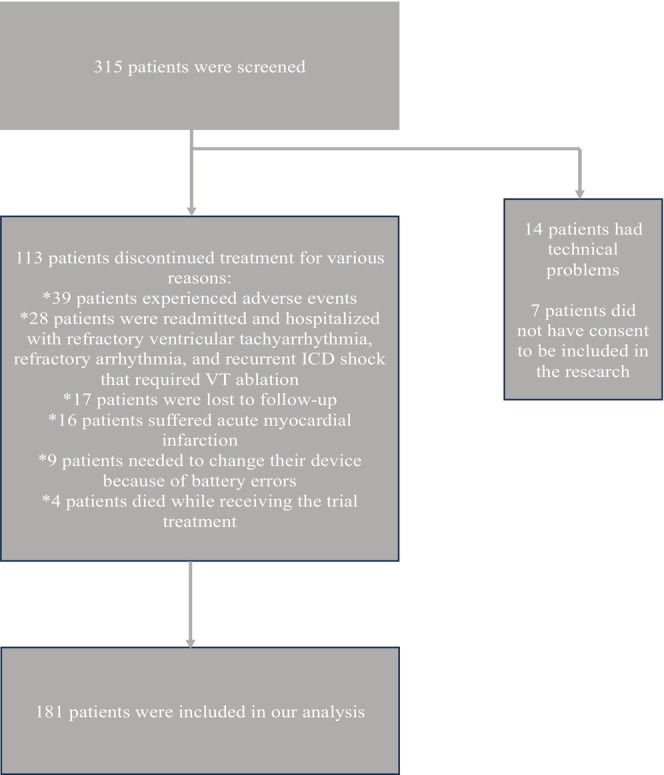
Screening and follow‐up of the population in our study.

From the initial cohort of 315 HFrEF patients with ICD or CRT‐D who were prescribed SV as an addition to their existing treatment, 113 patients were excluded due to discontinuation of treatment. Thirty‐nine patients experienced adverse events related to the study's treatment, 28 patients were readmitted and hospitalized with refractory ventricular tachyarrhythmia, refractory arrhythmia, and recurrent ICD shock requiring VT ablation, nine patients needed to change their device because of battery errors, 16 patients experienced acute myocardial infarction, 17 patients were missed to be followed, and four patients died during the follow‐up period (Figure 1). Ultimately, 181 HFrEF patients completed at least 12 months of follow‐up. The mean age of this group was 63.39 ± 12 years, with 36.5% being male. Among these patients, 60.8% had an ICD, while the remainder had CRT‐D. The characteristics of the study population are summarized in Table 1.

**TABLE 1:** Baseline characteristics before initiation of Sacubitril/Valsartan.

Variable	
Age (mean ± SD)	63.39 ± 12
Male (%)	36.5
NYHA (%)	
1	47
2	25.4
3	21.5
4	5.5
Smoking (%)	27.6
HTN (%)	39.8
DM (%)	30.9
Dyslipidemia (%)	38.1
CKD (%)	13.3
SBP (mean ± SD)	116.79 ± 21.74
HR (mean ± SD)	74.5 ± 14.28
HF (%)	
NICMP	30.4
DCM	24.86
NCC	2.2
PPCM	1.66
BOHCM	1.1
LDAC	1
ICMP	69.6
Duration of HF (mean ± SD) (years)	8.96 ± 6.79
Device (%)	
ICD	60.8
CRT‐D	39.2
Previous MI (%)	68
Previous stroke (%)	6.1
CABG (%)	26.5
Atrial fibrillation (%)	2.8
Digoxin (%)	27.1
LVEF	29.95 ± 9.61
LVEDD	61.39 ± 9.56

Abbreviations: BOHCM = burned‐out hypertrophic cardiomyopathy, CABG = coronary artery bypass grafting, CKD = chronic kidney disease, CRT‐D = cardiac resynchronization therapy‐device, DCM = dilated cardiomyopathy, DM = diabetes mellitus, HF = heart failure, HR = heart rate, HTN = hypertension, ICD = implantable cardioverter‐defibrillator, ICMP = ischemic cardiomyopathy, LDAC = left‐dominant arrhythmogenic cardiomyopathy, LVEDD = left ventricular end‐diastolic diameter, LVEF = left ventricular ejection fraction, MI = myocardial infarction, NCC = noncompaction cardiomyopathy, NICMP = non‐ischemic cardiomyopathy, NYHA = New York Heart Association, PPCM = peripartum cardiomyopathy, SBP = systolic blood pressure, SD = standardized deviation.

### 3.2 Outcomes

The differences in arrhythmia rates before and after treatment with SV demonstrated significantly improved results. The incidence of VF, assessed through device interrogation, revealed a notable decrease in VF (7 vs. 15), with a *p* = 0.025. However, the overall incidence of VT did not decrease during follow‐up. The combined outcome VT and VF, referred to as VTVF in our study, showed a significant reduction (17 vs. 24), with a *p* = 0.047. Similarly, the need for ICD interventions such as ATP and shocks significantly decreased after initiating SV treatment over a 12‐month follow‐up period (10 vs. 24), with a *p* = 0.012. Both outcomes showed independent significant reductions with *p*‐values of 0.043 and 0.041, respectively. Regarding echocardiography outcomes, LVEF improved significantly after 12 months of treatment with SV (*p* = 0.033), while LVEDD also decreased significantly (*p* = 0.047). Table 2 summarizes the differences in outcomes before and after the prescription of SV.

**TABLE 2:** Outcomes incidence before and after initiation of Sacubitril/Valsartan and *p*‐value of difference among follow‐up period of 12 months.

Outcomes	Pre‐S/V	Post‐S/V	*p*
SVT	6	11	0.185
NSVT	29	32	0.480
VT	12	14	0.400
VF	15	7	0.025
VTVF	24	17	0.047
ATP	14	10	0.043
Shock	14	6	0.041
Therapy	24	10	0.012
LVEF	29.95 ± 9.61	31.66 ± 10.44	0.033
LVEDD	61.39 ± 9.56	59.51 ± 9.92	0.047

Abbreviations: ATP = anti‐tachycardia pacing, LVEDD = left ventricular end‐diastolic diameter, LVEF = left ventricular ejection fraction, NSVT = non‐sustained ventricular tachycardia, S/V = Sacubitril/Valsartan, SVT = sustained ventricular tachycardia, VF = ventricular fibrillation, VT = ventricular tachycardia, VTVF = ventricular tachycardia + ventricular fibrillation.

### 3.3 Regression

Correlations between outcomes and demographic variables were conducted by using regression models (Table S1). A significant negative correlation was observed with a *p* = 0.047, indicating that the presence of DM is associated with lower VT incidence. Conversely, a positive relationship was found between ICD use and VT incidence, with a significant *p* = 0.034. The relationship between VF and NYHA4 was also significant, showing a positive correlation with a *p* = 0.014. Overall, the VTVF outcome, which stated for cumulative VT and VF incidences, had a significant positive relation with ICD and a significant negative relation with NHYA4, with *p*‐values of 0.023 and 0.030, respectively. Regarding treatment requirements, the association between ICD and therapy requirements was significant and positive, with a *p* = 0.040. The relationship between male gender is significant concerning LVEDD alterations and shock treatment, with *p*‐values of 0.033 and 0.044, respectively. Additionally, the associations are negative.

## 4 Discussion

SV, an angiotensin receptor‐neprilysin inhibitor (ARNI), stands out as one of the main treatments for patients diagnosed with HFrEF. Beyond its well‐established benefits in lowering hospitalizations and mortality, emerging evidence suggests that SV may also play an important role in attenuating the risk of ventricular arrhythmias (VAs), which could lead to SCD. This study demonstrates that SV therapy significantly reduces VTVF events and associated ICD interventions, indicating its potential to reduce the likelihood of lethal arrhythmic events and probable SCD. Additionally, we observed improvements in echocardiographic parameters, further enhancing the prognosis for these patients.

### 4.1 Reduction in Ventricular Arrhythmias

Along with our findings, several clinical studies have demonstrated that SV is associated with a reduction in the incidence of VAs in HF patients. A prospective cohort study by Diego et al. evaluating HFrEF patients with ICD, suggested that patients with SV therapy experienced fewer SVT episodes, sustained ventricular tachycardia, and appropriate ICD shocks in a significant way (de Diego et al. 2018). An observational study conducted by Martens et al. highlighted the advantages of SV over traditional ACE inhibitors or ARBs. It demonstrated that SV therapy had a meaningful association with the reduction of VT/VF episodes, which led to diminishing the need for therapeutic interventions. Moreover, the number of NsVT events was also reduced in the treatment with SV. However, SV therapy had no significant protective effect on the occurrence of AF (Martens et al. 2019). It was suggested that in patients with HFrEF, SV therapy could have an immense effect on reducing the usage of cardiac implantable devices, such as ICDs and CRT‐D, by improving LVEF and left ventricular reverse remodeling (Martin Dominguez et al. 2019; Belarte‐Tornero et al. 2021). Moreover, a similar observational study involving HFrEF patients with ICD demonstrated that SV therapy significantly reduced the incidence of both sustained and NSVT, as well as the necessity for ICD shocks (Gul et al. 2020). However, in a meta‐analysis of randomized control trials (RCTs) evaluating arrhythmia‐related outcomes, it was suggested that SV therapy did not significantly reduce the occurrence of Vas compared to control groups (Liu et al. 2022).

### 4.2 Impact on Overall Outcomes

Beyond its impact on VAs, previous large‐scale studies have illustrated that SV therapy has also been associated with lower rates of SCD, a leading cause of mortality in HF patients. The PARADIGM‐HF trial, a landmark double‐blind RCT, demonstrated a significant 20% reduction in cardiovascular death (including SCD and deaths related to worsening HF) when comparing SV to enalapril (McMurray et al. 2014). However, another RCT of 335 advanced HFrEF patients found no meaningful difference in all‐cause or cardiovascular mortality between those receiving SV and those on valsartan monotherapy (Mann et al. 2022). Further analysis from the PARADIGM‐HF trial revealed that SV therapy effectively reduced SCD rates across both ICD users and nonusers, besides improving overall survival and event‐free survival (Desai et al. 2015; Claggett et al. 2015). Another sub‐analysis of this prominent RCT conducted by Curtain et al. evaluated the risk of ventricular arrhythmia and the composite of this parameter with ICD shock and revived cardiac arrest, which showed that SV therapy significantly improved these outcomes in HFrEF patients (Curtain et al. 2022). Moreover, pooled analyses from PARAGON‐HF and PARADIGM‐HF evaluated the effect of SV therapy in different ranges of EF (Solomon et al. 2020). It indicated that while patients with an LVEF greater than 55% did not observe significant benefits, those within the 40%–50% range experienced lower rates of hospitalization and death. In the earlier mentioned meta‐analysis, it was demonstrated that patients with SV therapy have a 21% lower likelihood of experiencing SCD compared to the control groups (Liu et al. 2022). Aligning with the aforementioned studies, 2021 European guidelines for HF suggested SV as a therapeutic plan to decrease the rates of hospitalizations for HF and mortality (McDonagh et al. 2021). This therapy was also approved by the Food and Drug Administration and European Medicines Agency 2015 guidelines and is nowadays utilized in more than 100 countries (Abdin et al. 2022).

### 4.3 Improving the Echocardiographic Parameters

Furthermore, SV therapy has been associated with significant improvements in echocardiographic parameters, contributing to better patient prognoses. An RCT by Desai et al. suggested that patients receiving SV therapy experienced prominent improvements in echocardiographic metrics, such as left atrial volume, left ventricular end‐diastolic volume indices (LVEDVI), left ventricular end‐systolic volume indices (LVESVI), and E/e' ratio of mitral valve compared to those on enalapril (Desai et al. 2019). In an observational study by Casale et al. HFrEF patients with ICD or CRT‐D undergoing SV therapy observed similar results. The analysis suggested that patients experienced improvements in left ventricular reverse remodeling and EF (Casale et al. 2021). Another prospective observational study evaluating nonischemic dilated cardiomyopathy, also noted significant echocardiographic improvements, such as increasing LVEF and decreasing left ventricular dimensions, including LVEDD, left ventricular end‐systolic diameter (LVESD) and left atrial diameter (LAD) (Allam et al. 2024). In a real‐life observational study of patients with HFrEF, analysis demonstrated a meaningful reduction in left atrial volume index (LAVI), LVEDVI, LVESVI, right ventricular outflow tract (RVOT), and right atrial (RA) area, contrary to a meaningful improvement in functional echocardiography parameters, such as global longitudinal strain (GLS), LVEF, cardiac index, and Tricuspid annular plane systolic excursion (TAPSE) (Armentaro et al. 2021).

### 4.4 The Effect of Baseline Characteristics on the Outcomes

Our analysis identified factors such as DM, prior ICD usage, NYHA class IV status, and female gender as significant modifiers of SV therapy effects, including the occurrence of arrhythmia events. In line with our findings, a meta‐analysis conducted by Justine et al. suggested that women experienced less cardiac dysrhythmia compared to men undergoing pacemaker implantation procedures (Ravaux et al. 2021). Moreover, it was suggested that patients with higher NYHA classes typically have limited physical capability, which may affect their overall health and therapeutic efficacy (Zou et al. 2024). Those with diabetes often present with more severe symptoms and a higher rate of comorbidities, which can result in complicating disease management and affecting treatment responses (Nowakowska et al. 2019). These factors may contribute to the differences in patient responses to SV therapy, underscoring the importance of personalized therapeutic plans.

### 4.5 Mechanistic Insights Into AntiArrhythmic Effects

The mechanisms underlying the antiarrhythmic effects of SV remain an area of ongoing investigation. It was suggested that SV therapy could be a therapeutic plan for reducing the number of ventricular arrhythmias through three different pathways, including B‐type natriuretic peptide, angiotensin II, and bradykinin. Although the explicit process by which SV lowers the death rate due to malignant ventricular dysrhythmia is not yet clear, several studies have implicated its effect on reducing cardiac fibrosis as a key factor (Wei et al. 2022). SV appears to play an indirect protective role against arrhythmic events by modulating the expressions of ACE, GAV1, AGT, REN, and ADRB2 genes, which suppress the remodeling of cardiac tissue. Moreover, it suppresses the renin–angiotensin–aldosterone system (RAAS) pathway, effectively adjusting the vascular volume and tension (Zhou et al. 2022). Furthermore, the effect of SV therapy on gap junction remodeling, which is substantial in generating and preserving cardiac arrhythmias, could significantly diminish ventricular arrhythmias associated with myocardial hypertrophy (Nadarajah et al. 2021). Lastly, the regulatory effects of SV on cav1 and TP53, important biomarkers for initiating arrhythmia in the atherosclerosis pathway, may be a key factor in reducing the number of these adverse events (Zhou et al. 2022).

### 4.6 Limitations

Overall, this study contributes valuable insights into the impact of SV on cardiac function and arrhythmias in patients with HF. Although this study is one of the few articles evaluating HFrEF patients previously treated with ICT or CRT‐D regarding various outcomes utilizing robust statistical approaches, it had some notable limitations. One of the main limitations of this study, besides the observational design, is the retrospective view which poses inherent constraints. Moreover, the lack of a control group not receiving SV limits our capability to distinguish between the effect of this therapy and the natural recovery process following CRT‐D. Another concern is the limited number of participants, which could diminish the generalizability of our analysis to broader populations. The small sample of patients in our study, which results in a lower number of observed interventions, might reduce the reliability of our analyses. Throughout the follow‐up period, most of the patients did not reach the optimal SV dosage due to several considerations. Firstly, many patients complained about symptomatic hypotension, which negatively affected their daily activities. This finding was in line with a RCT that showed a high rate of symptomatic hypotension among patients on SV (Tsutsui et al. 2024) Furthermore, 60% of our patients did not have HTN, making the decrease in blood pressure of concern. Consequently, we were unable to increase the dosage of SV in these cases. Moreover, due to the 12‐month study duration, we prioritized evaluating arrhythmic events, as they are more readily observed in the short term. Therefore, we did not evaluate crucial end points, such as mortality or critical adverse clinical events. Lastly, the lack of measuring pro‐B‐type natriuretic peptide (pro‐BNP) consistently could affect the aforementioned reverse cardiac remodeling in our patients. Future research should involve RCTs with larger sample sizes and long‐term follow‐ups, focusing on populations with arrhythmias and appropriate controls, to explore major adverse cardiovascular outcomes, including hospitalization, stroke, HF, and mortality.

## 5 Conclusions

Patients taking SV as an adjunct therapy experienced a significant reduction in cardiac arrhythmias, such as VTVF, along with fewer clinical interventions for implanted devices. Simultaneously, there were notable improvements in echocardiographic parameters, which may serve as important clinical predictors of the prognosis for HFrEF patients.

## Author Contributions

The authors take full responsibility for this article.

## Ethics Statement

The Research and Ethics Committees of Shiraz University of Medical Sciences approved this study, which was done under the ethical principles outlined in the Declaration of Helsinki.

## Consent

Informed consent was obtained from all participants prior to their inclusion in this study.

## Conflicts of Interest

The authors declare no conflicts of interest.

## Supporting information


Table S1.

## Data Availability

The dataset employed in this research is available from the corresponding author upon a rational argument.
