# Supplementary material for: The Effect of Sacubitril/Valsartan on Supraventricular and Ventricular Arrhythmias in Patients With Heart Failure
Source: Ann Noninvasive Electrocardiol. 2025 Apr 15;30(3):e70081. doi: 10.1111/anec.70081 (PMC12000223; doi:10.1111/anec.70081)
Supplement: Supplementary file 1 — Table S1. [file ANEC-30-e70081-s001.docx]

**Supplementary material**

| Outcome | Gender | Age | NHYA4 | HTN | DM | CKD | Smoking | Digoxin | ICD | Duration of HF disease (year) |
| --- | --- | --- | --- | --- | --- | --- | --- | --- | --- | --- |
| SVT  Unstandardized beta  Standardized Coefficient beta  P-value | -0.494  -0.063  0.439 | 0.010  0.032  0.686 | 0.088  0.005  0.947 | 0.498  0.065  0.430 | -0.910  -0.112  0.161 | 0.801  0.073  0.390 | 0.373  0.045  0.578 | 0.761  0.009  0.255 | 0.068  0.009  0.910 | 0.019  0.035  0.659 |
| VF  Unstandardized beta  Standardized Coefficient beta  P-value | -.097  -0.040  0.617 | 0.008  0.083  0.285 | -0.996  -0.194  0.014 | -0.007  -0.003  0.972 | -0.067  -0.026  0.734 | 0.170  0.049  0.550 | -0.343  -0.131  0.095 | 0.247  0.093  0.226 | 0.245  0.101  0.184 | -0.005  -0.030  0.697 |
| VT  Unstandardized beta  Standardized Coefficient beta  P-value | -0.134  -0.027  0.732 | 0.011  0.057  0.456 | -1.166  -0.111  0.152 | -0.034  -0.007  0.930 | -0.795  -0.153  0.047 | 0.523  0.074  0.363 | 0.110  0.020  0.790 | 0.637  0.118  0.122 | 0.795  0.161  0.034 | 0.030  0.084  0.270 |
| VT/VF  Unstandardized beta  Standardized Coefficient beta  P-value | -0.230  -0.037  0.631 | 0.020  0.079  0.296 | -2.168  -0.167  0.030 | -0.041  -0.007  0.932 | -0.865  -0.135  0.077 | 0.689  0.079  0.326 | -0.230  -0.035  0.648 | 0.885  0.132  0.079 | 1.040  0.170  0.023 | 0.025  0.056  0.456 |
| NSVT  Unstandardized beta  Standardized Coefficient beta  P-value | -0.818  -0.088  0.272 | -0.012  -0.032  0.684 | -1.212  -0.063  0.431 | 0.627  0.069  0.394 | -0.808  -0.084  0.285 | 0.779  0.060  0.474 | 0.689  0.070  0.378 | 1.111  0.112  0.155 | -0.458  -0.050  0.516 | 0.060  0.091  0.246 |
| ATP  Unstandardized beta  Standardized Coefficient beta  P-value | 0.178  0.079  0.317 | 0.010  0.111  0.152 | -0.056  -0.012  0.879 | 0.210  0.096  0.233 | -0.182  -0.079  0.313 | -0.033  -0.011  0.898 | 0.087  0.036  0.642 | -0.024  -0.010  0.897 | 0.293  0.134  0.082 | -0.006  -0.036  0.647 |
| Shock  Unstandardized beta  Standardized Coefficient beta  P-value | -0.340  -0.162  0.044 | 0.010  0.116  0.139 | -0.018  -0.004  0.958 | 0.025  0.012  0.879 | -0.123  -0.057  0.472 | 0.157  0.053  0.521 | 0.163  0.073  0.357 | -0.021  -0.009  0.907 | 0.237  0.115  0.137 | 0.004  0.030  0.702 |
| Therapy  Unstandardized beta  Standardized Coefficient beta  P-value | -0.107  -0.035  0.655 | 0.018  0.152  0.051 | -0.092  -0.015  0.852 | 0.171  0.058  0.470 | -0.199  -0.064  0.413 | 0.054  0.013  0.877 | 0.202  0.063  0.421 | 0.085  0.026  0.734 | 0.468  0.158  0.040 | 0.008  0.039  0.610 |
| LVEF  Unstandardized beta  Standardized Coefficient beta  p-value | -0.177  -0.009  0.913 | -0.029  -0.036  0.649 | 4.289  0.102  0.203 | -0.946  -0.048  0.557 | -0.203  -0.010  0.902 | 1.714  0.060  0.471 | -2.392  -0.111  0.163 | -2.221  -0.102  0.194 | 0.436  0.022  0.777 | -0.165  -0.116  0.141 |
| LVEDD  Unstandardized beta  Standardized Coefficient beta  p-value | -3.631  -0.170  0.033 | 0.045  0.053  0.496 | -1.596  -0.036  0.649 | -2.953  -0.141  0.080 | -0.879  -0.040  0.610 | 1.834  0.061  0.459 | 0.537  0.024  0.763 | 1.211  0.053  0.495 | 0.823  0.039  0.608 | 0.096  0.063  0.413 |

Supplementary Table 1. Correlation between demographic variables and outcomes
